# Optimal hypertension screening frequency in adults: a systematic review

**DOI:** 10.1186/s12875-026-03403-5

**Published:** 2026-06-03

**Authors:** Valentin Daguisy, Nassir Messaadi, Judith Ollivon

**Affiliations:** 1 University of Lille, Lille, France; 2 Calais, France

**Keywords:** Hypertension, Screening, Blood pressure measurement, Primary health care

## Abstract

**Background:**

Blood pressure measurement for hypertension (HTN) screening is routine practice in primary care. The question remains regarding the optimal frequency of measurement to ensure accurate diagnosis while avoiding unnecessary repeated assessments.

**Aim:**

To determine the optimal frequency of HTN screening in adults.

**Methods:**

A systematic review was conducted in accordance with PRISMA guidelines. PubMed, Embase, Cochrane CENTRAL, and LiSSa databases were searched using standardized search strategies. The selection process was illustrated using a PRISMA 2020 flow diagram. Included studies were summarized in tables and qualitatively synthesized. The risk of bias of the included studies was assessed using the JBI critical appraisal tools. The initial search was conducted in April 2024, with an updated search performed in January 2026. To improve search sensitivity, an additional targeted search using controlled vocabulary and free-text terms was conducted in May 2026 in PubMed and Embase.

**Results:**

Two studies were identified through database searches: a prospective cohort study assessing blood pressure variability over time to determine the optimal screening interval for HTN, and a case–control study comparing the sensitivity and specificity of blood pressure measurement at each visit with those of annual measurement for HTN diagnosis. Six additional guideline publications were analyzed to provide an overview of existing international recommendations on this topic. Existing recommendations overall correspond to grade C-level recommendations, largely based on expert opinion.

**Conclusion:**

There is a paucity of primary research specifically addressing the optimal frequency of HTN screening. No study evaluating the impact of different screening frequencies on the incidence of HTN-related complications was identified. Available evidence is therefore limited and indirect.

## Introduction

Hypertension is one of the leading cardiovascular risk factors [1]. Screening enables early treatment and reduces the risk of HTN-related complications [2].

Blood pressure measurement is the procedure that enables HTN screening. The question arises as to the optimal frequency of measurement, in order to ensure appropriate diagnosis without performing unnecessary repeated assessments.

A 2020 Cochrane systematic review and meta-analysis [3] aimed to evaluate the effectiveness of different HTN screening strategies in reducing HTN-related morbidity and mortality. However, no eligible studies were identified.

Patients themselves hold perceptions regarding the appropriate frequency of blood pressure measurement. In this regard, a study [4] including 492 patients showed that 40.2% (95% CI: 35.9–44.6%) of patients in the context of an acute condition and 47.6% (95% CI: 41.8–53.3%) in chronic follow-up reported being bothered by the absence of blood pressure measurement.

Thus, in general practice, a substantial proportion of patients remain attached to the repetition of this procedure.

Blood pressure measurement may retain a strong symbolic dimension and likely plays an important role in the doctor–patient relationship. Among many physicians, this act is perceived as emblematic of the general practice consultation, and this perception may influence the frequency with which measurements are performed [5].

In this context, it appears important to explore the existing evidence specifically addressing the optimal frequency of blood pressure measurement strictly for HTN screening purposes.

The aim of this study was to determine the optimal frequency of blood pressure measurement for the screening of HTN in adults.

This work does not address blood pressure measurements performed to support diagnosis or assess severity in an acute clinical setting, as such measurements do not constitute HTN screening. Nor does it concern measurements performed in patients with known HTN, which fall within the scope of routine medical follow-up.

## Methods

### Study design

This study is a systematic review of the literature conducted in accordance with the PRISMA 2020 guidelines [6].

The review protocol was not prospectively registered in PROSPERO or another international registry. This work was initially conducted as a French medical degree thesis project, for which prospective registration is not routinely performed. However, the research question and planned methodology were predefined in a thesis proposal approved by the Faculty of Medicine of the University of Lille before the review was conducted.

### Databases

The databases searched were PubMed, Cochrane Central, Embase, and LiSSa. These databases were selected for their relevance, complementarity, and use of controlled vocabulary systems. LiSSa was included because of its coverage of French-language medical literature.

### Search strategies

The identification and selection of MeSH terms were performed using the HeTOP platform. Emtree terms were identified and selected from the Embase website.

A standardized search strategy was used with the MeSH and Emtree terms: “hypertension” and “screening.” The keywords “child,” “adolescent,” and “pulmonary hypertension” were used as exclusion terms.

Because the initial Embase query yielded broader and less specific results than the other databases, the additional Emtree terms “blood pressure measurement” and “blood pressure monitoring” were added and combined using the Boolean operator OR in order to improve specificity.

The detailed search strategies are provided in Appendix 3.

### Inclusion and exclusion criteria

#### Inclusion criteria

Randomized and non-randomized, prospective and retrospective studies allowing:

- comparison of the impact of two or more screening frequencies on cardiovascular morbidity and mortality or other health complications;
- provision of relevant evidence supporting one screening frequency over another or others.

#### Exclusion criteria

- studies not addressing screening in the context of ambulatory care;
- review articles (systematic reviews, meta-analyses, narrative reviews);
- clinical guidelines and expert-consensus recommendations.

### Selection process

The identification, screening, eligibility, and inclusion stages according to PRISMA 2020 were conducted independently by two investigators. The results were compared at the end of the selection process.

The planned mechanism for resolving potential disagreements consisted of discussion followed, if necessary, by arbitration.

Zotero software was used for article importation and organization.

The stages of the selection process were illustrated using a PRISMA 2020 flow diagram.

The initial selection process was conducted in April 2024. The second investigator conducted an independent selection process in December 2024. An updated search of the same databases using the same search strategies was performed in January 2026.

In response to reviewers’ comments regarding search sensitivity, an additional targeted search combining controlled vocabulary and free-text terms was subsequently conducted by one reviewer (VD) in May 2026 in the PubMed and Embase databases in order to identify potentially missed studies using alternative terminology.

### Data extraction and quality assessment of included studies

Data extraction was performed by one reviewer (VD). Data extracted from included studies included study design, participant characteristics, screening strategies, follow-up duration, evaluated outcomes related to HTN screening frequency, and main quantitative findings. Additional variables extracted when available included comparator strategies and study setting. All reported outcomes considered relevant to the research question were collected from the included studies. Reported effect measures included sensitivity, specificity, confidence intervals, and signal-to-noise ratios, depending on the included study design and outcomes. No assumptions were made regarding missing or unclear data.

Included studies were synthesized qualitatively according to their evaluated outcomes and methodological characteristics.

The methodological quality of included primary research studies was assessed using the Joanna Briggs Institute (JBI) critical appraisal tools [7], with the checklist selected according to the study design. Two reviewers independently conducted the appraisal (VD and JO), and disagreements were resolved through discussion. An overall appraisal of methodological quality and risk of bias was performed based on the relevance and number of criteria met.

### Data synthesis

Results were presented using descriptive tables. A quantitative synthesis was not planned because of the anticipated heterogeneity of study designs, evaluated outcomes, and methodological approaches. Therefore, a narrative qualitative synthesis of the included studies was performed.

Guideline publications identified through other sources were analyzed separately from the primary studies in order to provide contextual information regarding current international recommendations.

## Results

### Study selection process

Out of a total of 1,736 articles identified, 14 were selected after title and abstract screening. Two articles were retained for final inclusion after full-text review.

**Fig. 1:**
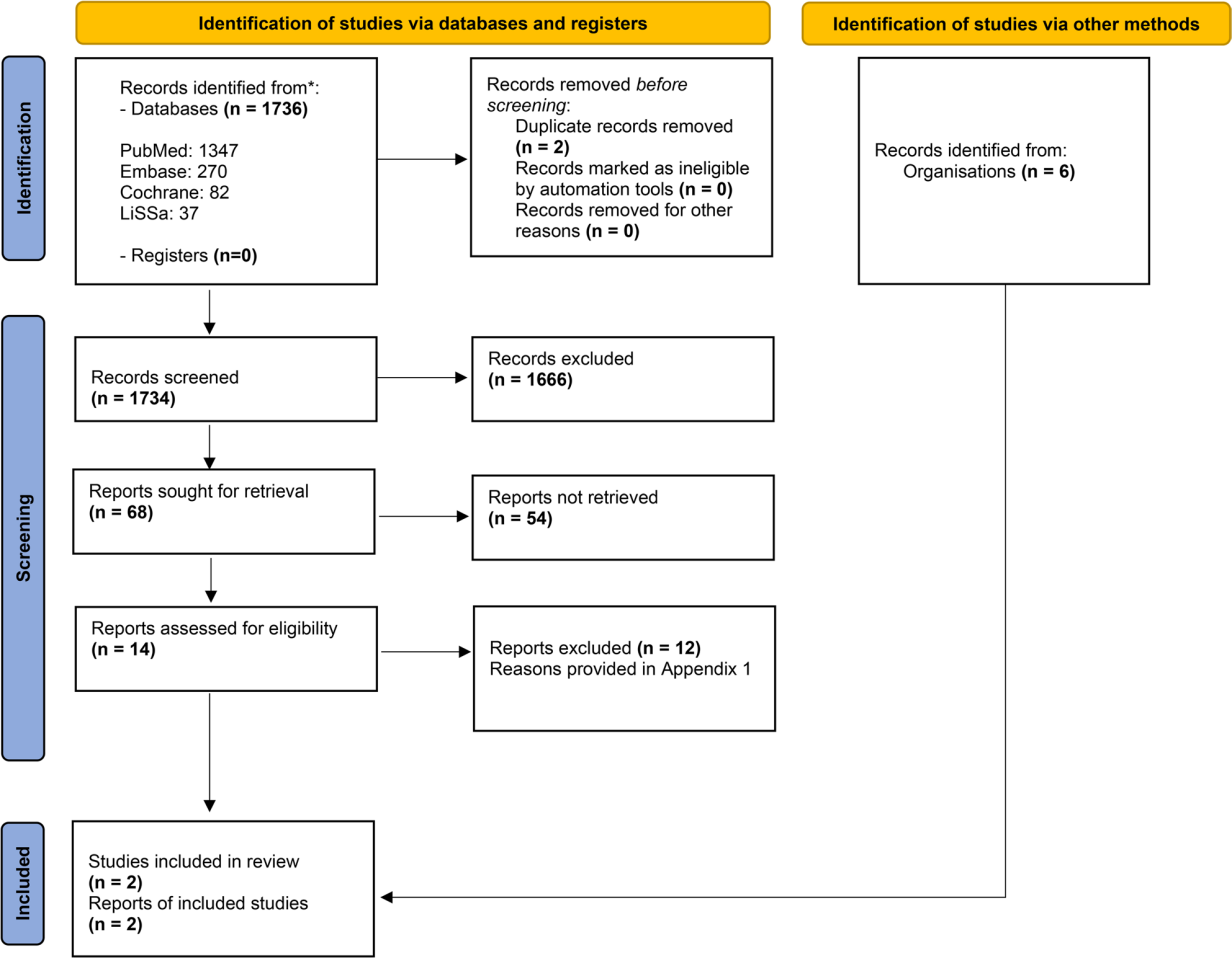
PRISMA 2020 flow diagram

The PRISMA flow diagram (Fig. 1) presents the initial study selection process from the databases.

The updated database search conducted in January 2026 identified seven additional articles after title screening. Five were excluded after abstract review, and the remaining two articles were excluded after full-text review.

The complementary targeted search conducted in May 2026 using controlled vocabulary and free-text terms identified five additional articles after title screening, duplicate removal, and exclusion of the two studies already included in the review. One article was excluded after abstract review, and the remaining four articles were excluded after full-text review.

The reasons for exclusion of studies after full-text review are detailed in Appendix 1. Their indirect contribution to the research question is discussed in the Discussion section when relevant.

### Included studies from databases

The characteristics of the included primary studies are presented in Table 1.

**Table 1:** Characteristics of the included primary studies

Author/ Year/Country	Method/Sample Size	PICO	Follow-up	Risk of Bias/Notable Limitations	Data Synthesis	Main Quantitative Results
Takahashi et al. [8] 2012 Japan	Prospective cohort study 15,055 individuals	Population: Healthy Japanese adults (> 20 years old) participating in an annual health check-up program at St Luke’s International Hospital (Tokyo), not receiving antihypertensive therapy at baseline. Intervention: Annual BP measurement over 3 years. Comparison: No comparison strategy Outcomes: Assessment of long-term BP change variance (“signal”) and short-term intra-individual variance (“noise”); signal-to-noise (S/*N*) ratio to estimate optimal screening interval and best BP parameter for HTN detection.	3 years	Selection bias: Participants recruited from a single hospital-based annual health check-up program. Attrition bias: Only 15,055 of the initial 36,866 participants completed all three annual measurements.	SBP and MAP showed the best screening performance (highest S/N ratio). Variability in SBP over time was greater in individuals with higher baseline SBP. The authors suggested that the optimal screening interval should be 3 years or more for individuals with SBP < 130 mmHg and 2 years for those with SBP ≥ 130 mmHg.	S/N ratio reached 1.0 for SBP and MAP at 3 years (lower at 1 and 2 years and for the other parameters). For SBP ≥ 130 mmHg: S/N > 1 at 2 years (S/N = 1.1); 1.6 at 3 years. For SBP < 130 mmHg: S/N < 1 at 3 years (S/N = 0.8).
Garrison et al.[9] 2013 USA	Retrospective case-control study 372 adults without HTN and 68 newly diagnosed hypertensive patients	Population: Adults aged 18–75 years followed in general practice at Mayo Clinic (Minnesota, USA). Intervention: Annual HTN screening strategy. Comparison: to usual practice (BP measured at each visit). Outcomes: Sensitivity and specificity of both screening strategies.	Retrospective study; data collected over the 5 years preceding August 1, 2010	Selection bias: Patients identified from an administrative database. Notable limitation: Patients with specific high-risk CV conditions were excluded, which may limit the applicability of the findings to populations most vulnerable to HTN-related complications.	Annual screening improved specificity without loss of sensitivity compared with usual practice.	Specificity improved from 70.4% (95% CI, 65.5%−75.0%) for the usual practice to 82.0% (95% CI, 77.7%−85.8%) for the annual screening strategy. No statistically significant difference in sensitivity existed between the 2 methods.

- ➢ Takahashi et al. (2012) [8].

This is a prospective cohort study. It assessed blood pressure variability over time in a cohort of 15,055 patients.

Its objective was to determine the most appropriate parameter (systolic blood pressure [SBP], diastolic blood pressure [DBP], mean arterial pressure [MAP], or pulse pressure [PP]) and the optimal screening interval for HTN.

To do so, the investigators determined the long-term true change variance in blood pressure (“signal”), corresponding to the actual long-term variation in blood pressure and the short-term within-person variance (“noise”), corresponding to variations related to short-term changing factors and not reflecting true long-term variation.

These data were used to determine the signal-to-noise ratio of blood pressure variations.

The findings showed that SBP and MAP appeared to be the most appropriate screening measures (higher signal-to-noise ratio compared with DBP and pulse pressure). Based on statistical modeling of the evolution of the signal-to-noise ratio over time according to baseline systolic blood pressure levels, the investigators suggested that the optimal HTN screening interval should be at least 3 years for individuals with a baseline SBP < 130 mmHg and every 2 years for those with a baseline SBP ≥ 130 mmHg.

Using the JBI checklist, the cohort study by O Takahashi et al. demonstrated several methodological strengths, including standardized outcome assessment, subgroup analyses, and appropriate statistical modeling, but limitations related to incomplete follow-up should be considered when interpreting the findings. Detailed JBI critical appraisal results for Takahashi et al. are presented in Appendix 2, Table 3. 

- ➢ Garrison et al. (2013) [9].

This is a retrospective case–control study conducted among 372 non-hypertensive patients and 68 patients newly diagnosed with HTN.

Its objective was to test the following hypothesis: a HTN screening strategy limited to annual measurement increases specificity while maintaining sensitivity.

To do so, the investigators compared the sensitivity and specificity of HTN detection between annual screening and usual screening practice based on blood pressure measurement at every visit.

The findings supported the study’s hypothesis: the significant increase in specificity resulting from a reduction in the number of measurements was not associated with a significant decrease in sensitivity.

Based on the JBI critical appraisal checklist, the case-control study by Garrison et al. used standardized diagnostic criteria and appropriate analyses to compare the diagnostic performance of two blood pressure screening strategies. However, important methodological limitations, including baseline differences between groups, absence of matching, and lack of adjustment for potential confounding factors, should be considered when interpreting the findings. Detailed JBI critical appraisal results for Garrison et al. are presented in Appendix 2, Table 4.

- ➢ Qualitative synthesis of included studies from databases

A quantitative synthesis was not feasible because of the limited number of included studies and the marked heterogeneity of their methodologies and evaluated outcomes.

The included studies did not directly compare the impact of different HTN screening frequencies on cardiovascular morbidity, mortality, or HTN-related complications. Instead, they explored distinct indirect parameters related to screening frequency. One study evaluated blood pressure variability and its signal-to-noise ratio over time in order to estimate rescreening intervals according to baseline systolic blood pressure levels, whereas the other assessed the diagnostic performance of annual screening compared with screening at every visit.

### Guideline articles

Six additional guideline publications identified through other sources were reviewed separately to provide an overview of current international recommendations on HTN screening frequency. These guidelines were issued by authorities or scientific societies from the United States, Europe, Canada, China, Japan, and the International Society of Hypertension.

These guideline publications recommend different screening frequencies according to baseline BP levels, HTN risk, cardiovascular risk, and age.

These different recommendations are presented in 2 and are associated with the grade of recommendation reported in the articles.

**Table 2:** Guideline articles identified through other sources

Organization/Country/Year	Target Population	Recommended Screening Frequency	Reported Grade
ESH Europe 2023 [10]	Adults	Depends on prior BP level, HTN risk, and CV risk	C
	Adults ≥ 40 years (or earlier in high-risk individuals)	Regular	C
	High-risk individuals (specific ethnic groups, high-normal BP, overweight/obese)	Annual	C
USPSTF USA 2021[11]	Adults ≥ 40 years or at increased risk (Black individuals, high-normal BP, overweight/obesity)	Annual	------
	Adults 18–39 years without increased risk and normal BP (< 130/85 mmHg)	Every 3–5 years	------
Canada 2020 [12]	Each patient’s risk of HTN Risk factors: diabetes mellitus, chronic kidney disease, low level of consumption of fresh fruits and vegetables and sedentary behavior	Can be tailored	------
	High-normal BP (SBP 130–139 or DBP 85–89 mmHg)	Annual	C
	Individuals without known HTN and no macrovascular target-organ damage	Annual	D(Canadian grading)
ISH International 2020 [13]	BP < 130/85 mmHg without CV risk factors	Every 3 years	------
	BP < 130/85 mmHg with CV risk factors	Annual	------
	BP ≥ 130/85 mmHg	Repeated office measurements or home/ABPM if possible	------
China 2024 [14]	« hypertension-prone populations »	Annual	------
	« high-risk hypertension groups »	At least every 6 months	------
	Key risk factors: high-normal BP, overweight/obesity, excessive alcohol consumption, a family history of HTN, high salt intake, age > 55 years or postmenopausal women		
JSH Japan 2025 [15]	Not explicitly specified	No explicit mention	

------: not provided

## Discussion

### Synthesis and comparison with existing data

The specific question of the optimal frequency of HTN screening has been little studied. The article selection process identified only two primary research studies: a cohort study (level of evidence 2), and a retrospective case–control study (level of evidence 3). No study compared the incidence of HTN-related complications among groups screened at different frequencies.

The available data to determine the most appropriate frequency of HTN screening are therefore currently scarce and indirect.

The scarcity of data highlighted here is consistent with that observed in the 2020 systematic review and meta-analysis conducted by Cochrane [3]. Its scope was broader, as it aimed to evaluate more generally the effectiveness of different HTN screening strategies. However, the investigators expected results based directly on clinical outcomes for patients or consequences for the healthcare system. No study met the inclusion criteria.

The study by Garrison et al., comparing annual HTN screening with more frequent screening, addresses a clinically relevant question. However, beyond the methodological limitations identified through the JBI appraisal, the exclusion of patients with several high-risk cardiovascular conditions may limit the interpretability and applicability of the findings, particularly for some populations at greater risk of HTN-related complications.

Besides the study by Takahashi et al., several longitudinal studies (Appendix 1: studies 13, 17, and 18) suggest that various factors influence the risk of developing HTN, particularly baseline blood pressure level, age, body mass index, and weight variation. These findings indirectly support the hypothesis that the most appropriate frequency of HTN screening may differ according to an individual’s baseline risk profile rather than relying on a uniform screening interval for the entire population. This individualized approach is broadly consistent with current international recommendations, which generally propose adapting HTN screening frequency according to baseline blood pressure levels and overall risk profile.

### Strengths and limitations

#### Strengths

To our knowledge, this is the only review specifically addressing the question of the optimal frequency of HTN screening.

The research question originates from a common and practical issue encountered in daily general practice.

Despite the scarcity of direct evidence, this review proposes a pragmatic synthesis intended to inform clinical practice while respecting the current limits of available knowledge.

#### Limitations

The main limitation of this work lies in the limited number of primary studies directly addressing the research question, which constrains the strength of the conclusions that can be drawn. Furthermore, the available evidence is based on indirect outcomes rather than on direct clinical endpoints (e.g., morbidity, mortality, or cardiovascular complications). This limits the clinical interpretability of the findings and the ability to synthesize results.

A complementary targeted search using controlled vocabulary and free-text terms was subsequently performed to improve search sensitivity. Nevertheless, the possibility remains that some relevant studies were not identified because of heterogeneity in terminology and indexing across databases.

Although the research question and methodology were predefined before the review was conducted, the absence of prospective protocol registration may have reduced the transparency and reproducibility of the review process.

### Considerations and perspectives for further research

The limited number of eligible studies identified, with no recent primary research over the past decade, warrants consideration given the widespread use of blood pressure measurement in primary care. Several factors may explain this gap. First, evaluating the optimal frequency of blood pressure measurement for screening purposes presents important methodological challenges, as it would require large, long-term studies with clinically relevant outcomes. Second, research has mainly focused on diagnostic methods and the management of established HTN rather than on screening strategies in asymptomatic populations. Third, current recommendations, often based on expert consensus, may have reduced the perceived need for further primary research. In addition, screening practices in routine care are frequently opportunistic and influenced by organizational factors, limiting their standardization within study protocols. Taken together, these elements may account for the lack of recent evidence.

Ideally, prospective studies comparing the incidence of HTN-related complications across randomized groups exposed to different screening frequencies would provide more robust evidence, although such designs may be challenging to implement in practice.

The increasing availability of wearable and connected blood pressure monitoring devices raises the question of whether the concept of a fixed screening frequency based on office measurements will remain relevant in the future. By enabling repeated or near-continuous measurements in real-life settings, these technologies may shift current approaches toward more flexible and patient-centered strategies. However, their role in population-level screening remains uncertain, given persistent challenges regarding measurement validity, standardization, and clinical integration [16].

In routine clinical practice, the frequency of blood pressure measurement is likely influenced by factors beyond the sole objective of HTN screening. Two elements appear important to explore in order to adapt daily practice in the most appropriate and integrative way: patients’ and physicians’ beliefs regarding the act of blood pressure measurement and the probable value of this act within the doctor–patient relationship, independently of the quantitative measurement per se.

## Conclusion

This systematic review highlights the scarcity of data regarding the optimal frequency of HTN screening.

The available primary research consists of studies that do not use HTN-related complications as outcome measures. These data are therefore indirect.

Existing recommendations on this topic are mainly based on low-level evidence and expert consensus.

### Proposal for a practical synthesis

Based on the currently available limited and indirect evidence and existing clinical guidelines, we propose the following pragmatic interpretation:

For the diagnosis of HTN, an annual screening strategy may not be less sensitive than more frequent screening; however, this finding is based on a single observational study excluding patients with several high-risk cardiovascular conditions and should be interpreted with caution.

The pattern of blood pressure variability observed in one prospective cohort study suggests that screening intervals of at least 3 years for individuals with a baseline SBP < 130 mmHg and every 2 years for individuals with a baseline SBP ≥ 130 mmHg may cautiously be considered; however, these results are derived from surrogate markers rather than clinical outcomes.

International guidelines generally recommend adapting screening frequency according to several factors, including baseline BP levels, HTN risk, cardiovascular risk, and age.

Overall, these elements should be interpreted as practice-informed and hypothesis-generating rather than evidence-based recommendations.

## Data Availability

All data generated or analysed during this study are included in this published article.

## Appendix

### Appendix 1: Characteristics of studies excluded after full-text review

*From the initial study selection process (April 2024)*

1. *Hodes C*,* Rogers PA*,* Everitt MG*.
  *Clinical science and molecular medicine. Supplement*,* 1976*,* 3*,* 657–659 s | added to CENTRAL: 30 April 1999 | 1999 Issue 2.*

Study evaluating the value of organized HTN screening in a specific age group on diagnosis and effective treatment of patients, compared with usual practice where physicians measure blood pressure depending on the consultation context.

The study shows that screening is beneficial. However, there is no evaluation of the optimal screening frequency.

2. *Bodenmann P*,* Pasche O*,* Jaunin-Stalder N*,* Willi C*,* Pasche C*,* Ombelli J*,* Cornuz J. Nouveautés en médecine ambulatoire : dépistage*,* traitement et iatrogénicité [Innovations in ambulatory care: screening*,* treatment and iatrogenicity]. Rev Med Suisse. 2008 Jan 30;4(142):289 − 94. French. PMID: 18,383,937.*

This article addresses target values in the management of HTN in older adults; it does not explore screening frequency.

3. *Ménard J. Les va-et-vient de l’histoire de l’hypertension artérielle. La Revue du praticien. 2010 Mai 20;60(5):638 − 43.*

Epidemiological article that does not include any evaluation of optimal screening frequency.

4. *Bernard Rosner*,* B. Frank Polk*,* predictive values of routine blood pressure measurements in screening for hypertension*,* American Journal of Epidemiology*,* Volume 117*,* Issue 4*,* April 1983*,* Pages 429–442*

Epidemiological study aimed at developing consistent screening rules that take blood pressure variability into account, particularly regarding the number of measurements required to establish a diagnosis while limiting misclassification errors.

Overall, the study determined that 93% of hypertensive and non-hypertensive individuals could be identified after three visits.

5. *Ferrer Soler C*,* Ehret G*,* Pechère-Bertschi A. Dépistage et prise en charge de l’hypertension artérielle chez la personne âgée [Screening and management of hypertension in elderly]. Rev Med Suisse. 2015 Sep 9;11(485):1638*,* 1640-4. French. PMID: 26,540,992.*

French-language article on the specific features of HTN in older adults, its screening, the value of home blood pressure monitoring, and the need for caution regarding orthostatic hypotension.

No evaluation of screening frequency.

6. *Louis A. De Nino*,* Cynthia D. Mulrow. Screening for Hypertension. Ann Intern Med.1990;112:796–797.*

This is the letters and corrections section of *Annals of Internal Medicine*, volume 112, issue 10, referring back to the 1990 article by Littenberg and Garber discussed below. It questions the cost–benefit analysis of HTN screening.

7. *Littenberg B*,* Garber AM*,* Sox HC Jr. Screening for hypertension. Ann Intern Med. 1990 Feb 1;112(3):192–202.*

Article exploring four questions related to HTN, each addressed in a separate section.

One section provides a modeled estimate of the cost–benefit ratio of HTN screening, but there is no comparison of two or more screening frequencies.

Another section discusses the issue of screening frequency and outlines unresolved questions necessary to address it, notably: what is the probability that a normotensive individual will develop HTN during the screening interval?

8. *Guirguis-Blake JM*,* Evans CV*,* Webber EM*,* Coppola EL*,* Perdue LA*,* Weyrich MS. Screening for Hypertension in Adults: Updated Evidence Report and Systematic Review for the US Preventive Services Task Force. JAMA. 2021 Apr 27;325(16):1657–1669.*

Objective: “To systematically review the benefits and harms of screening and confirmatory blood pressure measurements in adults, to inform the US Preventive Services Task Force.

Four questions were addressed: Does screening for HTN in adults improve health outcomes?; What is the accuracy of OBPM during a single encounter as initial screening for HTN compared with the reference standard (ABPM)?; What is the accuracy of confirmatory blood pressure measurement in adults who initially screen positive for HTN compared with the reference standard (ABPM)?; What are the harms of screening for HTN in adults?

Ultimately, the article does not address the question of optimal screening frequency.

9. *Abernethy JD. The Australian National Blood Pressure Study. Med J Aust. 1974 May 25;1(21):821-4. PMID: 4,605,365.*

Preliminary results of a study evaluating the impact of treating individuals screened and found to have moderate HTN, in terms of cardiovascular complications, within a controlled therapeutic trial.

The question of optimal screening frequency is not studied.

10. *Abdalla M*,* Muntner P*,* Peterson ED. The USPSTF Recommendation on Blood Pressure Screening: Making 2021 the Transformative Year in Controlling Hypertension. JAMA. 2021;325(16):1618–1619. *10.1001/jama.2021.4499.

Two-page summary reiterating the 2021 JAMA recommendations.

Includes discussion of appropriate HTN management and its particularities in the context of COVID-19.

11. *Van Buuren S*,* Boshuizen HC*,* Reijneveld SA. Toward targeted hypertension screening guidelines. Med Decis Making. 2006 Mar-Apr;26(2):145 − 53.*

Comparison of the cost-effectiveness of several HTN screening models.

However, no comparison of different screening frequencies.

12. *Anstey DE*,* Bradley C*,* Shimbo D. USPSTF Recommendation Statement on Hypertension Screening in Adults—Where Do We Go From Here? JAMA Netw Open. 2021;4(4):e214203.*10.1001/jamanetworkopen.2021.4203

Article commenting on the 2021 JAMA recommendation and the article by Guirguis-Blake et al., discussed above.

*From the updated search process (January 2026)*

13. *Tourdjman M*,* Jacobi D*,* Petit P*,* Vol S*,* Tichet J*,* Halimi J-M. Incidence à 10 ans de l’HTA dans la population générale: rôle des paramètres démographiques et cliniques*,* et implication pour la surveillance des normotendus. Arch Mal Coeur Vaiss. 2007 Août;100(8):615-9.*

Prospective cohort study aimed at evaluating the influence of risk factors on the occurrence of elevated blood pressure. It established the 10-year incidence of HTN in the cohort and showed an association between elevated blood pressure and the following factors: baseline blood pressure level, age, BMI; and in men: low daily walking level, alcohol consumption, and active smoking.

No comparison of two or more screening frequencies, and no sufficient elements to justify one screening frequency over another.

14. *Daroudi R*,* Sari AA*,* Zamandi M*,* Yousefi E. Economic evaluation of hypertension screening in Iran using a Markov model. PLoS One. 2025 Jul 22;20(7):e0303223. *10.1371/journal.pone.0303223.

This article is a medico-economic modeling study conducted in Iran. It evaluates the economic effectiveness of different HTN screening frequencies in the general population, comparing annual, biennial, and triennial strategies starting at age 30. Using a Markov model, the authors conclude that screening every 2 to 3 years from age 30 is the most cost-effective strategy in terms of cost per QALY gained.

However, this work is based on modeled data incorporating cardiovascular epidemiology, healthcare costs, and the organization of the Iranian healthcare system. The absence of observed clinical outcomes and the strong dependence on the local healthcare and economic context significantly limit the generalizability of these conclusions to other healthcare settings.

*From the complementary targeted search using controlled vocabulary and free-text terms (May 2026)*

15. *Nguyen T-P-L*,* Wright EP*,* Nguyen T-T*,* Schuiling-Veninga CCM*,* Bijlsma MJ*,* Nguyen T-B-Y*,* et al. (2016) Cost-Effectiveness Analysis of Screening for and Managing Identified Hypertension for Cardiovascular Disease Prevention in Vietnam. PLoS ONE 11(5): e0155699.*10.1371/journal.pone.0155699.

This study evaluated the cost-effectiveness of different hypertension screening strategies in Vietnam using a decision tree combined with a Markov model. Several screening intervals (one-off, annual, and biennial) and starting ages for screening were compared. The findings suggested that the economic performance of screening strategies varied according to age, sex, screening interval, and treatment coverage, with some biennial or targeted screening strategies appearing cost-effective under specific conditions.

Similarly to the Iranian medico-economic modeling study discussed above, this study was not included in the final analysis because its conclusions were derived from simulated modeling rather than from observed clinical outcomes associated with different hypertension screening frequencies. The results depended on multiple assumptions regarding epidemiological parameters, healthcare costs, treatment coverage, and healthcare system organization, which may limit the direct applicability and generalizability of the proposed screening intervals.

16. *Kaur G*,* Chauhan A*,* Prinja S et al.*,* Cost-effectiveness of population-based screening for diabetes and hypertension in India: an economic modelling study*,* The Lancet Public Health*,* 2021; 7*,* e65-e73*.

This medico-economic study evaluated the cost-effectiveness of multiple population-based screening strategies for diabetes and hypertension in India using a hybrid decision tree and Markov model. Different screening intervals ranging from annual screening to screening every 20 years were modeled, together with several assumptions regarding healthcare utilization and primary care organization. The study suggested that, under specific organizational conditions, screening every 3 to 5 years could become cost-effective, whereas annual screening could become cost-saving if access to primary care management substantially increased.

Similarly to the previously discussed Iranian and Vietnamese modeling studies, this article was not included in the final analysis because its conclusions were derived from simulated medico-economic projections rather than from observed clinical outcomes associated with different hypertension screening frequencies. The proposed screening intervals depended heavily on multiple modeling assumptions regarding epidemiology, healthcare utilization, treatment coverage, costs, and healthcare system organization, which limits the direct applicability of the proposed frequencies to the determination of an optimal hypertension screening interval.

17. *Bakx J*,* van den Hoogen H*,* van den Bosch W …*,* Development of Blood Pressure and the Incidence of Hypertension in Men and Women Over an 18-year Period*,* Journal of Clinical Epidemiology*,* 52*,* 531–538*

This study presents several elements indirectly relevant to the question of the optimal frequency of hypertension screening. Thanks to a particularly long longitudinal follow-up period (18 years), it showed that baseline diastolic blood pressure was a strong predictor of future hypertension risk. The findings also suggested that individuals with low baseline diastolic blood pressure and no weight gain during follow-up had a low risk of developing hypertension, whereas subjects with borderline diastolic blood pressure values (85–95 mmHg), particularly in the presence of weight gain, had an increased risk of developing hypertension. The authors therefore suggested that regular check-ups might not be necessary for certain low-risk individuals.

However, this study was not included in the final analysis because it did not directly compare different hypertension screening frequencies and was not designed to determine an optimal screening interval. Conclusions regarding the need for regular check-ups were based on an indirect interpretation of longitudinal blood pressure changes, without formal comparison between different screening strategies or evaluation of clinical outcomes associated with different measurement frequencies.

18. *Sung K*,* Kyung Yoo T*,* Yeon Lee M …*,* Appropriate screening interval to detect the development of chronic metabolic diseases*,* Diabetes Research and Clinical Practice*,* 2023; 199*.

This cohort study conducted in a large Korean population evaluated the time to development of several metabolic disorders, including hypertension, according to baseline metabolic parameters. The findings suggested that individuals with borderline blood pressure values developed hypertension more rapidly, leading the authors to propose individualized screening intervals.

However, this study was not included in the final analysis because it did not provide direct evidence allowing determination of the optimal frequency of hypertension screening. The proposed intervals resulted from an extrapolation basening strategies or any other robust approach supporting one screening frequency over another.

## Appendix 2

### Detailed JBI critical appraisal tables for included studies

**Table 3:** Methodological quality assessment of Takahashi et al. using the JBI critical appraisal tool for the cohort studies

JBI Item	Domain	Appraisal	Justification
Q1	Groups comparability	Not applicable	Single cohort of healthy adults recruited from the same screening program; no clearly distinct exposed/unexposed groups.
Q2	Exposure measurement consistency	Not applicable	The study did not include clearly distinct exposed and unexposed groups. However, blood pressure measurements were obtained uniformly using the same standardized protocol across participants.
Q3	Exposure validity and reliability	Yes	Standardized BP measurements performed by trained nurses using calibrated automatic devices; duplicate readings averaged.
Q4	Confounders identified	Yes	Potential confounding factors including dyslipidemia, diabetes, age, smoking and overweight were identified and explored.
Q5	Confounding management	Yes	Stratified and subgroup analyses were performed to assess the influence of potential confounding factors
Q6	Outcome absent at baseline	Not applicable	The study primarily evaluated longitudinal blood pressure variability and signal-to-noise ratio rather than the incidence of a clinical outcome such as hypertension.
Q7	Outcome measurement validity	Yes	Objective BP measurements obtained using validated standardized methods.
Q8	Follow-up duration adequacy	Yes	Three-year follow-up considered sufficient to assess BP variability and screening interval estimation.
Q9	Follow-up completeness	No	Follow-up was incomplete, and reasons for the large number of participants lost to follow-up were not clearly described.
Q10	Management of incomplete follow-up	Unclear	Last observation carried forward (LOCF) used for censored data: participants initiating antihypertensive treatment during follow-up, but no comprehensive strategy addressing overall incomplete follow-up was reported.
Q11	Statistical analysis	Yes	Appropriate statistical analyses were performed, including signal-to-noise ratio estimation and statistical modeling of its evolution over time according to baseline systolic blood pressure levels.
—	Overall appraisal	—	The study demonstrated several methodological strengths, including standardized outcome assessment, subgroup analyses, and appropriate statistical modeling, but limitations related to incomplete follow-up should be considered when interpreting the findings.

**Table 4:** Methodological quality assessment of Garrison et al. using the JBI critical appraisal tool for case-control studies

JBI Item	Domain	Appraisal	Justification
Q1	Group comparability	No	Cases and controls differed in age and BMI.
Q2	Matching	Unclear	Cases and controls were recruited from the same primary care source population and study period; however, no formal individual or frequency matching strategy was reported.
Q3	Case-control identification criteria	Yes	Cases and controls were identified using standardized ICD-9 diagnostic criteria combined with chart review.
Q4	Exposure measurement validity	Yes	Blood pressure measurements were obtained using calibrated devices and standardized clinical procedures.
Q5	Exposure measurement consistency	Yes	Blood pressure measurements were obtained using the same procedures in cases and controls.
Q6	Confounders identified	Yes	Differences in age and BMI between groups were reported and discussed.
Q7	Confounding management	No	No matching, stratification, or multivariable adjustment strategy was reported to control for potential confounding factors.
Q8	Outcome assessment validity	Yes	Hypertension status and blood pressure measurements were assessed using standardized clinical criteria, chart review, and calibrated devices in both groups.
Q9	Exposure period adequacy	Yes	The 5-year retrospective observation period was considered sufficient to evaluate hypertension detection strategies.
Q10	Statistical analysis	Yes	Appropriate statistical analyses were performed, including sensitivity and specificity estimation with confidence intervals.
—	Overall appraisal	—	The study used standardized diagnostic criteria and appropriate analyses to compare the diagnostic performance of two blood pressure screening strategies. However, important methodological limitations, including baseline differences between groups, absence of matching, and lack of adjustment for potential confounding factors, should be considered when interpreting the findings.

## Appendix 3

### Search strategies

*From the initial study selection process and update (April 2024 and January 2026)*

*On PubMed and Cochrane CENTRAL:*

(hypertension[MeSH Terms]) AND (screening[MeSH Terms]) NOT ((child[MeSH Terms] OR adolescent[MeSH Terms] OR pulmonary hypertension[MeSH Terms])) Activation of “humans” and “adult” filters on PubMed.

*On Embase:*

'hypertension'/de AND 'screening'/de AND ('blood pressure measurement'/de OR 'blood pressure monitoring'/de) NOT 'child'/de NOT'adolescent'/de NOT 'pulmonary hypertension'/de Activation of “humans”, “adult”, “aged“ and “very elderly“ filters.

*On LiSSa:*

hypertension (keywords only) AND mass screening (keywords only)

*From the complementary targeted search using controlled vocabulary and free-text terms (May 2026)*

*On PubMed:*

("Hypertension"[MeSH Terms] OR"hypertension"[Title/Abstract]OR "high blood pressure"[Title/Abstract]) AND ("Mass Screening"[MeSH Terms] OR"screening"[Title/Abstract] OR "case finding"[Title/Abstract] OR "preventive examination"[Title/Abstract] OR "opportunistic screening"[Title/Abstract]) AND ("screening interval*"[Title/Abstract] OR "screening frequency"[Title/Abstract] OR "frequency of screening"[Title/Abstract] OR "rescreen*"[Title/Abstract] OR "repeat screening"[Title/Abstract]) NOT ("Pulmonary Hypertension"[MeSH Terms] OR child[MeSH Terms] OR adolescent[MeSH Terms])

Activation of “humans” and “adult” filters.

*On Embase:*

('hypertension'/de OR hypertension:ti,ab OR 'high blood pressure':ti,ab) AND ('mass screening'/de OR screening:ti,ab OR 'case finding':ti,ab OR 'preventive examination':ti,ab OR 'opportunistic screening':ti,ab) AND ('screening interval*':ti,ab OR 'screening frequency':ti,ab OR 'frequency of screening':ti,ab OR rescreen*:ti,ab OR 'repeat screening':ti,ab) NOT ('pulmonary hypertension'/de OR 'child'/de OR'adolescent'/de)

Activation of “humans”, “adult”, “aged“ and “very elderly“ filters.

## Appendix 4

### Triangulation and guidelines update

*Triangulation*

The identification and selection process conducted by the second investigator showed a difference in the number of initially identified articles (1,819 versus 1,736). This is due to the time interval between the two selection processes (April 2024 for the first investigator and December 2024 for the second).

The second investigator’s database selection process identified 7 articles for inclusion, compared with 2 for the first investigator.

These 7 articles included the 2 already included. The remaining 5 consisted of 4 articles also identified in the first investigator’s selection process but excluded at the full-text review stage (reasons detailed in Appendix 1) and 1 two-page reference consisting of a multiple-choice questionnaire and its answers based on a clinical case. The answers were based on the 2021 US Preventive Services Task Force guidelines, which were included as a guideline article from other sources.

*Guidelines update*

The latest 2024 publication of the ESH [17] and the 2025 Canadian recommendations [18] do not provide explicit elements regarding the optimal frequency of HTN screening. For the other sources, the most recent available version was used and presented in the corresponding table (Table 2).

## Abbreviations

BP: Blood Pressure
HTN: Hypertension
SBP: Systolic Blood Pressure
DBP: Diastolic Blood Pressure
MAP: Mean Arterial Pressure
CV: Cardiovascular
